# Cardiac deceleration capacity and acceleration capacity have diagnostic value in patients with vasovagal syncope regardless of age

**DOI:** 10.3389/fcvm.2024.1495129

**Published:** 2024-12-18

**Authors:** Jijing Wang, Jinyi Xu, Yanyan Qiu, Ruike Yang, Wentao Wang, Chuanyu Gao

**Affiliations:** ^1^ Department of Cardiopulmonary Function, Zhengzhou University People's Hospital (Henan Provincial People's Hospital), Zhengzhou, Henan, China; ^2^ Department of Cardiopulmonary Function, Fuwai Central China Cardiovascular Hospital, Zhengzhou, Henan, China; ^3^ Department of Cardiology, Fuwai Central China Cardiovascular Hospital, Zhengzhou, Henan, China; ^4^ Department of Cardiology, Zhengzhou University People's Hospital (Henan Provincial People's Hospital), Zhengzhou, Henan, China

**Keywords:** deceleration capacity, acceleration capacity, vasovagal syncope, autonomic nervous function, syncope

## Abstract

**Background:**

Deceleration capacity (DC) and acceleration capacity (AC) are used to characterize autonomic regulation. The purpose of this study was to evaluate the autonomic nervous function in patients with vasovagal syncope (VVS) and to evaluate the diagnostic value of DC and AC for VVS.

**Methods:**

A total of 94 consecutive patients with VVS [51.0 (38.0–60.0) years; 48 males] and 76 healthy subjects [53.0 (44.3–62.8) years; 46 males] were recruited as controls. The study compared DC, AC, and heart rate variability (HRV) in 24-h ECG, echocardiogram, and biochemical examinations between the two groups.

**Results:**

DC was significantly higher (9.3 ± 2.1 vs. 7.4 ± 1.4 ms, *p* < .001) and AC was lower (−9.3 ± 2.1 vs. −7.3 ± 1.3 ms, *p* < .001) in the syncope group compared to the control group. HRV indicators were higher in the syncope group. In multivariable analyses, DC [odds ratio = 1.746 (95% CI, 1.389–2.195); *p* < .001], AC [odds ratio = 0.553 (95% CI, 0.435–0.702); *p* < .001] were independently associated with syncope. Mean HR was associated with syncope only in patients <60 years of age. Receiver operating characteristics (ROC) curves showed areas under curve (AUC) of DC/AC for predicting syncope are 0.755/0.765 with sensitivity of 56.4%/60.6% and specificity of 93.4%/88.2%.

**Conclusion:**

Patients with VVS exhibit higher DC and lower AC. Both DC and AC are independently correlated with syncope. A DC value >9.0 ms and an AC value −9.0 ms could potentially be valuable indicators for monitoring cardiac autonomic nervous dysfunction.

## 1 Introduction

Syncope is defined as a brief episode of unconsciousness and Vasovagal syncope (VVS) is the most prevalent type of syncope (1, 2). VVS is characterized by sudden onset and spontaneous recovery, usually with a benign course. However, frequent episodes can significantly impact quality of life and increase the risk of complications (3). Studies have shown that patients with VVS tend to have a lower quality of life compared to control populations, with factors such as gender, age, and frequency of syncopes correlating with this decline (4). Additionally, VVS is associated with an elevated risk of physical injury, particularly concerning for individuals in high-risk occupational settings (5). The exact pathophysiological mechanisms of VVS are still not fully understood (6).

Cardiovascular autonomic dysfunction plays a key role in the pathophysiology of VVS. VVS is thought to be closely linked to the dysregulation of autonomic nerves, leading to an imbalance between sympathetic and vagal nerves (3, 7). While a diagnosis of VVS can often be based on the patient's medical history in the presence of typical triggers, it may not always be feasible to rely solely on historical information (1).

The head-up tilt-table test (HUT) is crucial for identifying the underlying causes of unexplained syncope. However, some patients may find the tilt testing uncomfortable and challenging to maintain in an upright position (8, 9).

Traditional measurement of heart rate variability (HRV) has been used to analyze the function of the cardiac autonomic nervous system. Previous studies have presented conflicting results regarding VVS. HRV analysis is a well-established method for evaluating beat-to-beat neural heart rate modulation and its changes in various diseases. It can be challenging to distinguish the effects of vagal and sympathetic modulators on the heart for analysis (10, 11).

Deceleration capacity (DC) and acceleration capacity (AC) of heart rate (HR) have been developed as quantitative measures to evaluate cardiac vagal and sympathetic function (12, 13). DC has been validated as a predictor of mortality in patients following a myocardial infarction (12), while AC has been identified as a predictor for exacerbation of heart failure in patients with dilated cardiomyopathy (14).

A decrease in cardiac DC is indicative of reduced vagal tone in cardiac autonomic function. Previous studies have indicated that individuals with VVS exhibit higher DC values compared to control groups. A DC value greater than 7.5 ms could potentially be a useful tool for monitoring cardiac vagal activity and distinguishing VVS (5). However, existing research has mainly focused on young patients, with a gap in literature exploring the relationship between AC and VVS. Therefore, our study aims to investigate the impact of autonomic nervous system (ANS) function, as assessed by DC and AC, on both VVS and control groups, irrespective of age.

## 2 Methods

### 2.1 Ethical approval

The study was approved by the Ethics Committee of Henan Provincial People's Hospital (2018 Ethics Review No. 24) prior to conducting performance and clinical investigations, adhering to the principles outlined in the Declaration of Helsinki.

### 2.2 Study population

We included 94 consecutive patients [51.0 (38.0–60.0) years; 48 males] who were admitted to the Henan Provincial People's Hospital with suspected VVS. All patients underwent a tilt table test (TTT) as part of their diagnostic assessment. VVS was diagnosed based on clinical characteristics suggestive of a reflex mechanism and after excluding other differential diagnoses (5, 15). Exclusion criteria comprised individuals with a history of overt heart failure, myocardial infarction, left ventricular ejection fraction less than 50%, significant valvular heart disease, dilated cardiomyopathy, hypertrophic cardiomyopathy, sinus node dysfunction, arrhythmogenic right ventricular cardiomyopathy, Brugada syndrome, Long QT syndrome, and symptomatic orthostatic hypotension. Seventy-six healthy subjects [53.0 (44.3–62.8) years; 46 males] were enrolled as controls. They had undergone normal routine physical examinations and had never reported syncopal episodes in the past. Additionally, all subjects had normal 24-h Holter monitoring and echocardiograms.

### 2.3 Holter recording

A Holter monitor test was performed on each patient using a portable electrocardiogram device (CONTEC Medical System LTD, Qinhuangdao, China). The test measured various indices included average, fastest and slowest HR, deceleration capacity, acceleration capacity and HRV. HRV included standard deviation of normal-to-normal (NN) intervals (SDNN), standard deviation average of NN intervals (SDANN), root mean square successive difference of normal R-R intervals (RMSSD), and the percent of the number of times that the difference between adjacent normal RR intervals >50 ms in the total number of NN intervals (PNN50). The SDNN and SDANN were considered as measured of vagal and sympathetic influences, while RMSSD and PNN50 were regarded as indicators of parasympathetic nerve activity (16).

### 2.4 Deceleration capacity and acceleration capacity

The heart rate deceleration and acceleration capacities were measured by the Holter system (5). Calculation methods as previous described: Firstly, heart beat intervals were selected as decelerating anchors when >1.00 but ≤1.05 of the preceding heartbeat interval; heart beat intervals were selected as accelerating anchors when <1.00 but ≥0.95 of the preceding heartbeat interval. Secondly, the segments of heartbeat intervals around decelerating and accelerating anchors were collected. Thirdly, the above segments were aligned at the decelerating and accelerating and the signals of segments were averaged to obtain the phase-rectified signal averaging signal X(i).

In the end, the following formula was used to quantify deceleration capacity (DC) and acceleration capacity (AC): DC/AC=[X(0)+X(1)−X(−1)−X(−2)]/4.

The values of DC are over 0, quantifying vagal nerve activity and the values of AC are less than 0, quantifying sympathetic nerve activity (17).

### 2.5 Echocardiographic evaluation

In our study, all patients underwent transthoracic echocardiography (TTE) using a Sonos 5500 Ultrasound machine (Philips). The following parameters measured by the M-mode technique: right atrial diameter (RAd) and left atrial diameter (Lad), left ventricular end-diastolic diameter (LVEDd), and left ventricular end-systolic diameter (LVESd). Simpson's biplane method was used to measure the left ventricular ejection fraction (LVEF).

### 2.6 Statistical analysis

Continuous data are presented as mean ± SD or median (25th–75th percentile). Continuous variables were compared using a Student *t*-test for normally distributed data or a Wilcoxon ranksum test otherwise. Categorical variables were presented as sample size (percentage) and were compared using a Pearson *χ*^2^ test. multivariate logistic regression were employed to assess associations among various variables of interest with syncope. The receiver operator characteristics curve was performed to test the best cutoff value and the area under curve (AUC) of DC and AC to differentiate VVS and controls. All statistical analyses were two-sided, and a *P*-value < 0.05 was considered statistically significant.

## 3 Results

### 3.1 Comparison between syncopal patients and controls

There were no significant differences in gender, body mass index (BMI), echocardiogram, biochemical examinations and HR (all *p* > .05). DC was significantly higher (9.3 ± 2.1 vs. 7.4 ± 1.4 ms, *p* < .001) and AC was significantly lower (−9.3 ± 2.1 vs. −7.3 ± 1.3 ms, *p* < .001) in patients with VVS compared with controls. The HRV parameters including SDNN, RMSSD and PNN50 were increased in VVS group compared with controls (*p* < .05). Clinical characteristics of both groups are summarized in Table 1.

**Table 1:** Baseline patient characteristics between patients with controls and VVS.

	Control group (*n* = 76)	VVS patients (*n* = 94)	F/Z/*X^2^*	*p* value
Gender, male/female, *n*	46/30	48/46	1.522	0.217
Age, years	53.0 (44.3–62.8)	51.0 (38.0–60.0)	−1.805	0.071
BMI, kg/m^2^	24.1（22.6–25.1)	23.7 (22.7–24.6)	−1.233	0.217
Fasting glucose, mmol/L	4.8 ± 0.6	4.9 ± 0.8	0.700	0.204
Serum creatinine, umol/L	64.7 ± 12.9	64.1 ± 11.2	2.458	0.747
SBP, mmHg	120.0 ± 11.4	121.7 ± 12.0	0.076	0.240
DBP, mmHg	75.7 ± 8.4	77.4 ± 7.6	1.589	0.181
LAD, mm	32.4 ± 4.1	32.1 ± 4.5	0.481	0.634
LVEDD, mm	45.8 ± 2.9	45.0 ± 2.8	0.468	0.068
LVEF, %	65.7 ± 4.0	65.1 ± 3.6	0.047	0.384
DC, ms	7.4 ± 1.4	9.3 ± 2.1	18.826	**<0** **.** **001**
AC, ms	−7.3 ± 1.3	−9.3 ± 2.1	18.674	**<0** **.** **001**
SDNN, ms	115.0 ± 22.1	126.6 ± 31.5	8.001	**0** **.** **007**
SDANN, ms	114.4 ± 28.4	114.8 ± 29.2	0.086	0.769
RMSSD, ms	44.5 ± 18.3	53.3 ± 18.7	0.019	**0** **.** **002**
PNN50, %	5.9 ± 4.5	8.3 ± 5.3	2.563	**0** **.** **002**
Mean HR, bpm	73.2 ± 9.8	73.7 ± 8.5	0.953	0.727
Minimum HR, bpm	53.6 ± 6.4	53.1 ± 6.0	0.120	0.606
Maximum HR, bpm	113.5 ± 18.9	116.3 ± 18.3	0.007	0.326

VVS, vasovagal syncope; BMI, body mass index; SBP, Systolic blood pressure; DBP, diastolic blood pressure; LAD, left atrial diameter; LVEDD, left ventricular end diastolic diameter; LVEF, left ventricular ejection fraction; DC, deceleration capacity; AC, acceleration capacity; SDNN, standard deviation of normal-to-normal intervals; SDANN, standard deviation average of normal-to-normal intervals; RMSSD, root mean square successive difference of normal R-R intervals; PNN50,the percent of the number of times that the difference between adjacent normal RR intervals >50 ms in the total number of NN intervals; HR, heart rate.

Values in bold indicate statistical significance (*p* < .05).

### 3.2 Comparison of DC and HRV parameters between VVS patients and controls in different age groups

There are 114 participants <60 years of age (45 in the control group and 69 in the VVS group) and 56 participants ≥60 years of age (31 in the control group and 25 in the VVS group). Table 2 shows that in patients with VVS, the DC and absolute value of AC were significantly higher compared to controls, regardless of age.

**Table 2:** DC and HRV characteristics with controls and VVS in different age.

	Control group (<60 years)（*n* = 45)	Control group （≥60 years） （*n* = 31)	VVS patients (<60 years) （*n* = 69)	VVS patients （≥60 years) （*n* = 25)
DC, ms	7.7 ± 1.4	7.0 ± 1.3*	9.4 ± 2.0**	9.1 ± 2.2***
AC, ms	−7.6 ± 1.3	−6.9 ± 1.3*	−9.4 ± 2.1**	−9.0 ± 2.3***
SDNN, ms	114.8 ± 22.5	115.2 ± 21.9	127.8 ± 30.6**	123.1 ± 34.6
SDANN, ms	112.9 ± 26.1	116.5 ± 31.8	118.2 ± 29.7	105.5 ± 26.2
RMSSD, ms	45.5 ± 17.9	43.0 ± 19.2	54.0 ± 17.5**	51.3 ± 21.9
PNN50,%	6.3 ± 4.9	5.3 ± 4.1	8.9 ± 5.4**	6.7 ± 4.5
Mean HR, bpm	71.5 ± 8.6	75.6 ± 11.0	75.6 ± 8.2**	68.4 ± 6.9***^,^ ****
Minimum HR, bpm	52.8 ± 6.6	54.6 ± 6.1	53.1 ± 6.2	53.1 ± 5.6
Maximum HR, bpm	112.6 ± 18.1	114.8 ± 20.2	121.4 ± 16.7**	102.3 ± 15.0***^,^ ****

VVS, vasovagal syncope; DC, deceleration capacity; AC, acceleration capacity; SDNN, standard deviation of normal-to-normal intervals; SDANN, standard deviation average of normal-to-normal intervals; RMSSD, root mean square successive difference of normal R-R intervals; PNN50, the percent of the number of times that the difference between adjacent normal RR intervals >50 ms in the total number of NN intervals; HR, heart rate.

* Compared with control group (<60 years), *p* < 0.05.

** Compared with control group (<60 years), *p* < 0.05.

*** Compared with control group (>60 years), *p* < 0.05.

**** Compared with VVS patients (<60 years), *p* < 0.05.

However, SDNN, RMSSD, and PNN50 differed between VVS and control groups only in participants <60 years of age. The mean HR and maximum HR were higher in the VVS group compared to controls in participants <60 years of age, lower in participants ≥60 years of age. Interestingly, the DC and absolute value of AC were lower in control groups across different age groups, while there was no significant difference in DC and absolute value of AC in VVS groups across different age groups

### 3.3 Prediction of syncope in different age groups

The univariate regression analysis (Supplementary Table 1) showed that DC, AC, SDNN, RMSSD, and PNN50 were associated with VVS. However, the results of the multivariable regression analyses indicated that only DC [odds ratio = 1.746 (95% CI, 1.389–2.195); *p* < .001] and AC [odds ratio = 0.553 (95% CI, 0.435–0.702); *p* < .001] were significantly correlated with syncope (Table 3).

**Table 3:** The relationships between VVS and deceleration capacity, acceleration capacity in the entire cohort.

DC model			AC model		
Variables	*p* value	OR (95% CI)	Variables	*p* value	OR (95% CI)
DC	**<0** **.** **001**	1.746 (1.389–2.195)	AC	**<0** **.** **001**	0.553 (0.435–0.702)
SDNN	0.416	1.006 (0.991–1.021)	SDNN	0.648	1.003 (0.989–1.018)
RMSSD	0.655	1.006 (0.981–1.032)	RMSSD	0.670	1.006 (0.980–1.032)
PNN50	0.452	0.961 (0.868 −1.065)	PNN50	0.559	0.970 (0.875–1.075)

DC, deceleration capacity; AC, acceleration capacity; SDNN, standard deviation of normal-to-normal intervals; RMSSD, root mean square successive difference of normal R-R intervals; PNN50, the percent of the number of times that the difference between adjacent normal RR intervals >50 ms in the total number of NN intervals; OR, odds ratio.

Values in bold indicate statistical significance (*p* < .05).

In participants under 60 years of age, the univariate regression analysis indicated that DC, AC, SDNN, RMSSD, PNN50, mean HR, and maximum HR were correlated with VVS (Supplementary Table 2). Furthermore, the multivariable regression analyses demonstrated that not only DC and AC, but also mean HR (odds ratio = 1.111, 95% CI 1.018–1.213; *p* = .000), were significantly linked to syncope (Table 4).

**Table 4:** The relationship between VVS and deceleration capacity, acceleration capacity in <60 years of age.

DC model			AC model		
Variables	*p* value	OR (95% CI)	Variables	*p* value	OR (95% CI)
DC	**0** **.** **007**	1.485 (1.115–1.976)	AC	**0** **.** **002**	0.621 (0.460–0.839)
SDNN	0.097	1.019 (0.997–1.041)	SDNN	0.098	1.019 (0.997–1.042)
RMSSD	0.556	1.010 (0.977–1.044)	RMSSD	0.636	1.008 (0.975–1.043)
PNN50	0.982	1.001 (0.884 −1.134)	PNN50	0.945	0.996 (0.877–1.130)
Mean HR	**0** **.** **019**	1.111 (1.018–1.213)	Mean HR	**0** **.** **011**	1.124 (1.028–1.229)
Maximum HR	0.602	0.990 (0.954–1.028)	Maximum HR	0.471	0.986 (0.949–1.024)

DC, deceleration capacity; AC, acceleration capacity; SDNN, standard deviation of normal-to-normal intervals; RMSSD, root mean square successive difference of normal R-R intervals; PNN50, the percent of the number of times that the difference between adjacent normal RR intervals >50 ms in the total number of NN intervals; OR, odds ratio; HR, heart rate.

Values in bold indicate statistical significance (*p* < .05).

In participants aged 60 years and above, the univariate regression analysis showed correlations between VVS and DC, AC, mean HR, and maximum HR (Supplementary Table 3). Further multivariable regression analyses revealed that only DC and AC were significantly associated with syncope (Table 5).

**Table 5:** The relationship between VVS and deceleration capacity, acceleration capacity in ≥60 years of age.

DC model			AC model		
Variables	*p* value	OR (95% CI)	Variables	*p* value	OR (95% CI)
DC	**0** **.** **003**	1.956 (1.258–3.042)	AC	**0** **.** **003**	0.498 (0.314–0.790)
Mean HR	0.162	0.932 (0.845–1.029)	Mean HR	0.240	0.944 (0.857–1.039)
Maximum HR	0.574	0.987 (0.941–1.034)	Maximum HR	0.331	0.977 (0.932–1.024)

DC, deceleration capacity; AC, acceleration capacity; HR, heart rate; OR, odds ratio.

Values in bold indicate statistical significance (*p* < .05).

### 3.4 Receiver operator characteristic curve for prediction of VVS with DC and AC

ROC analysis was utilized to assess the predictive value of DC and AC in determining VVS. The AUC values for DC/AC in predicting syncope were 0.755/0.765, with sensitivities and specificities of 56.4%/60.6% and 93.4%/88.2% respectively at a cutoff of 9.4 ms/−9.0 ms.

**Figure 1:**
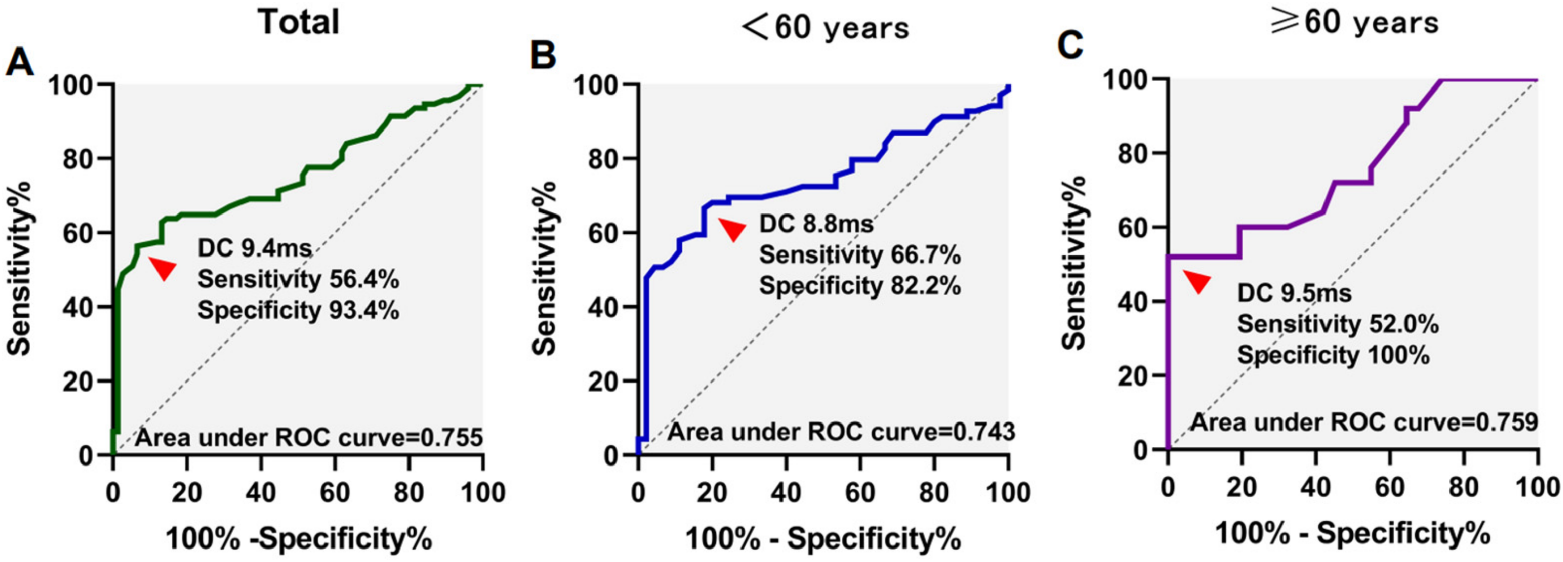
Receiver operating characteristics (ROC) curves for deceleration capacity (DC) for differentiation of syncope. ROC curves in total patients **(A)**, <60 years of age **(B)**, and ≥60 years of age **(C)** patients were showed.

**Figure 2:**
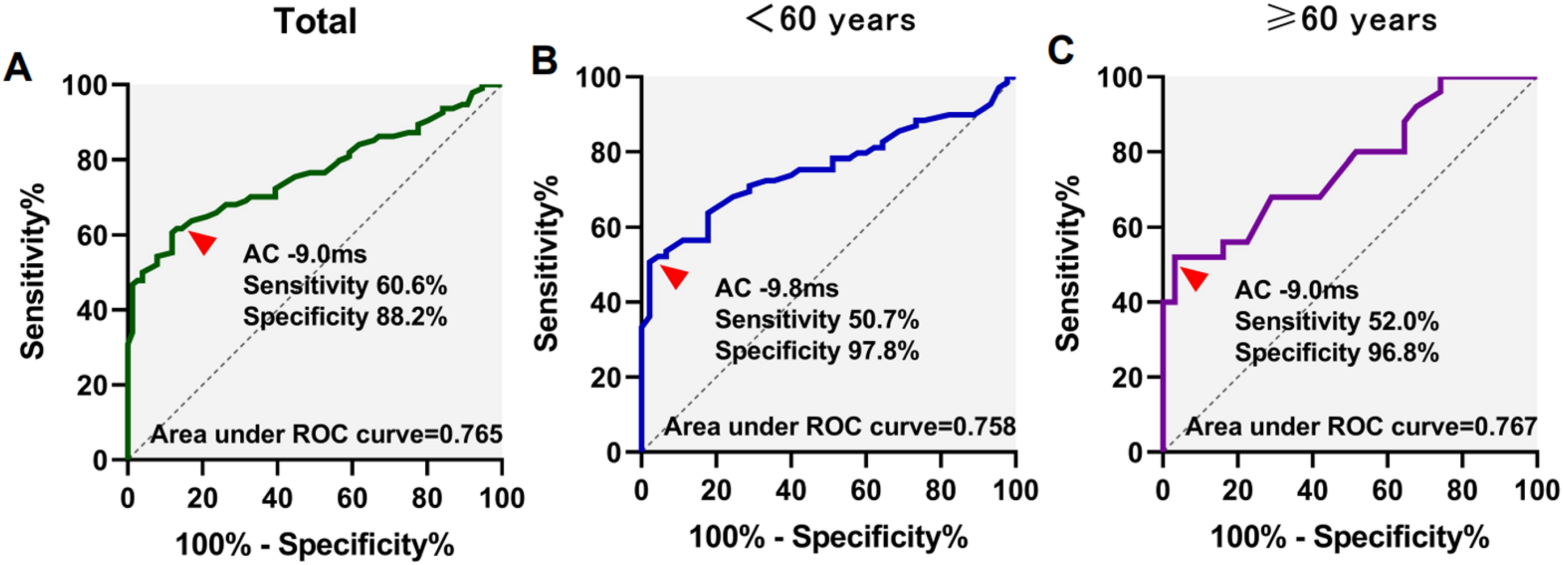
Receiver operating characteristics (ROC) curves for acceleration capacity (AC) for differentiation of syncope. ROC curves in total patients **(A)**, <60 years of age **(B)**, and ≥60 years of age **(C)** patients were showed.

In participants under 60 years of age, the area under the curve (AUC) for DC/AC predicting syncope was 0.743/0.758, with a sensitivity and specificity of 66.7%/50.7% and 82.2%/97.8% at the cutoff of 8.8 ms/−9.8 ms, respectively. For participants aged 60 years or above, the AUC for DC/AC predicting syncope was 0.759/0.767, with a sensitivity and specificity of 52.0%/52.0% and 100%/96.8% at the cutoff of 9.5 ms/−9.0 ms (Figures 1, 2). The AUC was similar for DC, AC, DC combined with HRV index, and AC combined with HRV index (Supplementary Table 4). In Supplementary Figure 1, the ROC curves almost completely overlap.

## 4 Discussion

The mechanisms involved in VVS have not been fully elucidated. In the research of VVS, focus has been on the role of autonomic nerves. It is believed that VVS is directly associated with altered regulatory functions of autonomic nerves, leading to an imbalance between sympathetic and vagal nerves (3, 7).

Sympathetic nervous system activity serves as a rapid regulatory system that enables the cardiovascular system to adapt to postural changes. Previous research has indicated that the vasodilation observed during vasovagal syncope (VVS) is due to a decrease in sympathetic tone, with sympathetic control of total peripheral resistance being the primary mechanism of VVS (18, 19).

Hypotension and/or bradycardia were observed during VVS, with bradycardia primarily mediated by parasympathetic means through the vagal nerve. Histological studies of the human heart have shown a higher presence of parasympathetic nerves compared to sympathetic nerves in the atrium (20). Recent research suggests that selective vagal denervation in the atrial subendocardium through catheter ablation could potentially prevent VVS recurrence (21, 22). Therefore, heightened vagal activity plays a significant role in the pathophysiology of VVS.

Tilt table testing has been recognized as a valuable diagnostic tool for VVS, although it does have certain limitations. The sensitivity and specificity of TTT are not optimal, with the test yielding positive results in only 78%–92% of patients meeting the clinical diagnostic criteria for VVS. Additionally, some individuals may experience discomfort, particularly during a positive TTT result (8, 9, 23, 24).

The effects of vagal and sympathetic modulators on the heart can be challenging to differentiate for analysis. Various studies have examined whether patients with VVS exhibit differences in baseline autonomic tone compared to healthy controls, with conflicting results. Some studies indicate that patients with VVS have heightened vagal autonomic tone, while others propose the opposite (10, 25). HRV reflects the integrated changes in autonomic functions controlled by both sympathetic and vagal regulation, without isolating the vagal component. Additionally, HRV is influenced by various factors (10).

DC and AC are innovative indicators of the autonomic nervous system. They utilize a signal processing algorithm to distinguish between deceleration and acceleration of heart rate, serving as a metric for cardiac autonomic nervous modulation. DC and AC offer advantages over traditional techniques like TTT and HRV. Firstly, they enable a quantitative assessment of autonomic activity in patients with VVS. Secondly, DC and AC values, calculated through phased-rectified signal averaging, are less susceptible to noise interference and demonstrate superior sensitivity, specificity, and stability compared to HRV (5, 12).

Our study revealed abnormally increased vagal tone, as assessed by DC, and decreased sympathetic activation, as assessed by AC, in patients with VVS compared to healthy controls. The findings suggest that the heightened baseline vagal regulation, along with sympathetic nervous system modulation during upright posture, may contribute to these patients being more susceptible to bradycardia, hypotension, and ultimately syncope.

Aging is known to affect autonomic function and responses to head-up tilt (HUT) in patients with syncope. Studies have shown changes in serum catecholamine levels during HUT testing, with younger fainters (<40 years) exhibiting higher Epi/NE ratios. Variations in clinical characteristics and response patterns to head-up tilt have been observed between young (≤35 years) and older (≥65 years) patients, suggesting potential differences in the underlying pathophysiological mechanisms (26). The frequency of cardioinhibitory response decreases with age, possibly due to increased vagal activity in younger patients compared to older individuals (27). Previous studies have found that DC was increased in VVS, but this was only applicable to young patients (<60 years) (5).

In our study, we found that DC was lower and AC was higher in VVS patients. This indicates vagal tone withdrawal and increased sympathetic activity in VVS patients, irrespective of age.

## 5 Conclusion

The absolute values of AC and DC were found to be higher in patients with VVS compared to the control group. Both AC and DC were identified as independent correlation factors for VVS. These findings suggest that DC and AC have diagnostic significance not only for younger VVS patients, but also for older individuals with VVS.

### 5.1 Study limitations

This study has several limitations. Firstly, there were only 94 patients with VVS included in our study, and more prospective studies are needed to investigate the association between deceleration and acceleration capacities with VVS patients. Secondly, we did not specifically exclude conditions such as diabetes, hypertension, obesity, and chronic lung disease, which might have an impact on autonomic activity. For instance, cardiovascular autonomic neuropathy (CAN) is an under-recognized yet highly prevalent microvascular complication of diabetes, affecting approximately 20% of those with the condition. However, the enrolled patients were relatively healthy, and few demonstrated the above conditions. Third, further detailed, larger sample, multicenter and longitudinal studies may be required in the future.

## Data Availability

The raw data supporting the conclusions of this article will be made available by the authors, without undue reservation.
