# Supplementary material for: Cardiac deceleration capacity and acceleration capacity have diagnostic value in patients with vasovagal syncope regardless of age
Source: Front Cardiovasc Med. 2024 Dec 18;11:1495129. doi: 10.3389/fcvm.2024.1495129 (PMC11688274; doi:10.3389/fcvm.2024.1495129)
Supplement: Supplementary file 1 [file Datasheet1.pdf]

Supplementary Material

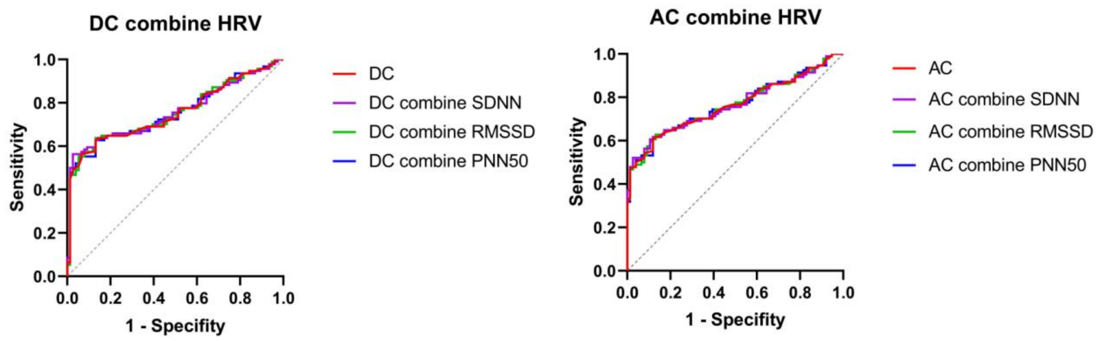

Supplementary Figure 1. Receiver operating characteristics(ROC) curves for DC and AC combine HRV for differentiation of syncope.

ROC curves in DC combine HRV index such as (left), and AC combine HRV index (right) were showed.

Supplementary Table 1. Univariate and multivariate regression analysis of DC, AC, HRV and syncope.

	univariate regression		multivariate regression	
Variables	***p*** value	**OR (95% CI)**	***p*** value	**OR (95% CI)**
**DC**	**＜0.001**	**1.743（1.429-2.125）**	**＜0.001**	**1.746（1.389-2.195）**
**AC**	**＜0.001**	**0.553（0.448-0.682）**	**＜0.001**	**0.553（0.435-0.702）**
**SDNN**	**0.009**	**1.106（1.004-1.028）**		
**SDANN**	0.919	**1.001（0.990-1.011）**		
**RMSSD**	**0.003**	**1.026（1.009-1.044）**		
**PNN50**	**0.003**	**1.105（1.034 - 1.181）**		

Mean HR	0.726	1.006 （0.973 - 1.040）
Minimum HR	0.603	1.105（0.940 - 1.037）
Maximum HR	0.324	1.008 （0.992 - 1.025）

Note: Values in bold indicate statistical significance ($p < .05$).

Abbrevations：DC, deceleration capacity; AC, acceleration capacity; SDNN, standard deviation of normal-to-normal intervals; SDANN,standard deviation average of NN intervals; RMSSD, root mean square successive difference of normal R-R intervals; PNN50,the percent of the number of times that the difference between adjacent normal RR intervals > 50 ms in the total number of NN intervals; OR, odds ratio;HR, heart rate.

Supplementary Table 2. Univariate and multivariate regression analysis of DC, AC, HRV and syncope in ＜60 years of age

	univariate regression		multivariate regression	
Variables	***p*** value	**OR (95% CI)**	***p*** value	**OR (95% CI)**
**DC**	**＜0.001**	**1.629（1.290-2.057）**	**0.007**	**1.485（1.115-1.976）**
**AC**	**＜0.001**	**0.587（0.458-0.753）**	**0.002**	**0.621（0.460-0.839）**
**SDNN**	**0.019**	**1.018（1.003-1.033）**		
**SDANN**	0.330	**1.007（0.993-1.021）**		
**RMSSD**	**0.016**	**1.028（1.005-1.052）**		
**PNN50**	**0.015**	**1.104（1.020 - 1.196）**		
Mean HR	**0.015**	1.064 （1.012 - 1.118）	**0.019**	**1.111（1.018-1.213）**

Minimum HR	0.850	1.006（0.947 - 1.068）		
Maximum HR	**0.011**	1.031 （1.007 - 1.055）		

Note: Values in bold indicate statistical significance ($p < .05$).

Abbrevations：DC, deceleration capacity; AC, acceleration capacity; SDNN, standard deviation of normal-to-normal intervals; SDANN,standard deviation average of NN intervals; RMSSD, root mean square successive difference of normal R-R intervals; PNN50,the percent of the number of times that the difference between adjacent normal RR intervals > 50 ms in the total number of NN intervals; OR, odds ratio;HR, heart rate.

Supplementary Table 3. Univariate and multivariate regression analysis of DC, AC, HRV and syncope in ≥60 years of age.

	univariate regression		multivariate regression	
Variables	***P*** value	**OR (95% CI)**	***P*** value	**OR (95% CI)**
**DC**	**0.001**	**1.963（1.314-2.935）**	**0.003**	**1.956（1.258-3.042）**
**AC**	**0.004**	**0.501（0.330-0.762）**	**0.003**	**0.498（0.314-0.790）**
**SDNN**	0.304	**1.010（0.991-1.030）**		
**SDANN**	0.174	**0.987（0.968-1.006）**		
**RMSSD**	0.137	**1.021（0.994-1.048）**		
**PNN50**	0.237	**1.080（0.951 - 1.226）**		
Mean HR	**0.011**	0.911 （0.848 - 0.979）		
Minimum HR	0.333	0.955（0.871 - 1.048）		
Maximum HR	**0.019**	0.959 （0.926 - 0.993）		

Note: Values in bold indicate statistical significance ($p < .05$).

Abbrevations：DC, deceleration capacity; AC, acceleration capacity; SDNN, standard deviation of normal-to-normal intervals; SDANN,standard deviation average of NN intervals; RMSSD, root mean square successive difference of normal R-R intervals; PNN50,the percent of the number of times that the difference between adjacent normal RR intervals > 50 ms in the total number of NN intervals; OR, odds ratio;HR, heart rate.

Supplementary Table 4. The predicting efficacy of cardiac DC and AC in combination with HRV

	**Area under ROC curve**	***p* value**
**DC**	**0.755**	**＜0.001**
**AC**	**0.765**	**＜0.001**
**SDNN**	**0.613**	**0.011**
**RMSSD**	**0.636**	**0.002**
**PNN50**	**0.639**	**0.002**
**DC+SDNN**	**0.758**	**＜0.001**
**DC+RMSSD**	**0.756**	**＜0.001**
**DC+PNN50**	**0.756**	**＜0.001**
**AC+SDNN**	**0.765**	**＜0.001**
**AC+RMSSD**	**0.765**	**＜0.001**
**AC+PNN50**	**0.767**	**＜0.001**

Note: Values in bold indicate statistical significance ($p < .05$).

Abbrevations：DC, deceleration capacity; AC, acceleration capacity; SDNN, standard deviation of normal-to-normal intervals; RMSSD, root mean square successive difference of normal R-R intervals; PNN50,the percent of the number of times that the difference between adjacent normal RR intervals > 50 ms in the total number of NN intervals; ROC,Receiver operating characteristics.
